# Effect of Microbiome on Non-Alcoholic Fatty Liver Disease and the Role of Probiotics, Prebiotics, and Biogenics

**DOI:** 10.3390/ijms22158008

**Published:** 2021-07-27

**Authors:** Mayumi Nagashimada, Masao Honda

**Affiliations:** Division of Health Sciences, Graduate School of Medical Science, Kanazawa University, Kanazawa 920-0942, Japan; nakanaga@staff.kanazawa-u.ac.jp

**Keywords:** NAFLD, NASH, insulin resistance, inflammation, fibrosis, dysbiosis, intestinal permeability

## Abstract

Non-alcoholic fatty liver disease (NAFLD) is a leading cause of liver cirrhosis and hepatocellular carcinoma. NAFLD is associated with metabolic disorders such as obesity, insulin resistance, dyslipidemia, steatohepatitis, and liver fibrosis. Liver-resident (Kupffer cells) and recruited macrophages contribute to low-grade chronic inflammation in various tissues by modulating macrophage polarization, which is implicated in the pathogenesis of metabolic diseases. Abnormalities in the intestinal environment, such as the gut microbiota, metabolites, and immune system, are also involved in the pathogenesis and development of NAFLD. Hepatic macrophage activation is induced by the permeation of antigens, endotoxins, and other proinflammatory substances into the bloodstream as a result of increased intestinal permeability. Therefore, it is important to understand the role of the gut–liver axis in influencing macrophage activity, which is central to the pathogenesis of NAFLD and nonalcoholic steatohepatitis (NASH). Not only probiotics but also biogenics (heat-killed lactic acid bacteria) are effective in ameliorating the progression of NASH. Here we review the effect of hepatic macrophages/Kupffer cells, other immune cells, intestinal permeability, and immunity on NAFLD and NASH and the impact of probiotics, prebiotics, and biogenesis on those diseases.

## 1. Introduction

Nonalcoholic fatty liver disease (NAFLD) is one of the most common chronic liver disorders worldwide and its prevalence is increasing [1,2]. NAFLD is characterized by hepatic damage caused by steatosis without secondary causes (e.g., medications, excessive alcohol consumption, or certain heritable conditions), which can progress to non-alcoholic steatohepatitis (NASH), fibrosis, cirrhosis, and hepatocellular carcinoma [3]. NASH has several histological features, such as steatosis, hepatocellular ballooning, lobular inflammation, and fibrosis [4,5].

**Figure 1:**
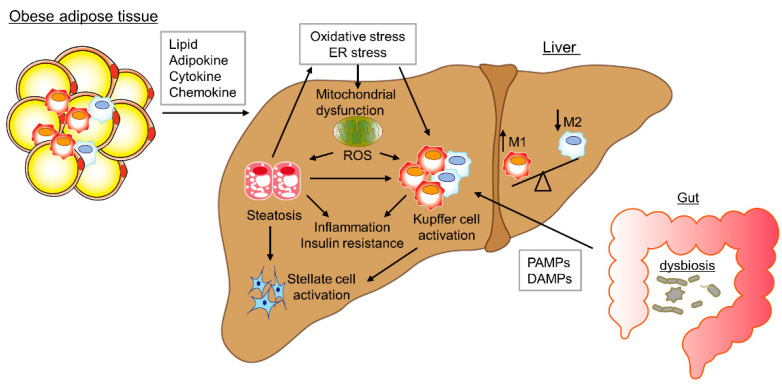
Multiple parallel-hit hypothesis of the progression of NAFLD/NASH. Overnutrition or inactivity caused by adipocyte hypertrophy and dysfunction. Obese adipose tissue shows chronic inflammation and insulin resistance as a result of infiltration of activated macrophages and T cells. Adipokines, such as interleukin-6 and tumor necrosis factor-α, and chemokines including CCL2 produced by adipocytes induce hepatocyte fat accumulation, hepatic inflammation, and insulin resistance. Overloading of triglycerides, free fatty acids, and free cholesterol induces endoplasmic reticulum stress and oxidative stress, leading to hepatic inflammation, mitochondrial dysfunction, fibrogenesis and, ultimately, hepatic steatosis. By contrast, microbial dysbiosis results in production of pathogen-associated molecular patterns and damage-associated molecular patterns, inducing an inflammatory response in hepatocytes, Kupffer cells, and hepatic stellate cells via a Toll-like receptor cascade, resulting in liver damage.

Several cross-sectional clinical studies have focused on the pathogenesis of NASH. In addition, mouse models of NASH mimic the pathogenesis of diet-induced obesity and its resulting metabolic disturbances, including NAFLD and NASH [6,7,8,9,10]. The development and progression of NAFLD is closely related to metabolic syndrome, insulin resistance, and type 2 diabetes mellitus (T2DM) [11,12,13,14,15,16]. NAFLD is a genetic-environmental-metabolic stress-related disease of unclear pathogenesis. The two-hit hypothesis explains progression of NASH from NAFLD. The first hit is insulin resistance and excessive fatty acids, which induce simple hepatic steatosis. The second hit is oxidative stress, lipid peroxidation, reactive oxygen species (ROS), and mitochondrial dysfunction. Moreover, recent findings support the multiple-hit hypothesis of the pathogenesis of NAFLD, which implicates liver, intestinal tract, and adipose tissue changes [17,18,19] (Figure 1). It is important to assess the role of inflammatory immune cells and oxidative stress in inducing inflammation, or of overproduction of ROS in liver mitochondrial dysfunction, a major pathogenic factor in NASH [20,21]. Dysregulation and polarization of macrophages (inflammatory M1 macrophages and anti-inflammatory M2 macrophages) in the liver contribute to the development and progression of NAFLD and NASH [22,23,24]. Oxidative stress-induced endoplasmic reticulum (ER) stress leads to upregulated liver lipogenic sterols, resulting in hepatic steatosis. Moreover, the activation of redox-sensitive nuclear factor-κB (NF-κB) by oxidative stress causes an increase in the expression of tumor necrosis factor-α (TNF-α), interleukin (IL)-1β and IL-6 [25]. The impaired mitochondrial respiratory chain caused by oxidative stress and peroxidation of cardiolipin (a dimeric phospholipid in the inner mitochondrial membrane) increases mitochondrial ROS production and promotes mitochondrial damage [26]. In addition, the intestinal barrier, immune cells, and microbiota composition are also involved in NAFLD and NASH development [27,28,29,30]. Herein we discuss the pathogenesis of NAFLD and NASH, and the potential of probiotics, prebiotics, biogenics for treatment.

## 2. Relationship between Hepatic Immune Cells and the Pathogenesis of NAFLD and NASH

Innate immune responses of NAFLD and NASH involve resident Kupffer cells, recruited macrophages derived from bone marrow cells, neutrophils, and natural killer T cells. These cells contribute to the progression of NASH by inducing inflammation, by promoting the production of cytokines, chemokines, eicosanoids, nitric oxide, and ROS. In addition, excessive free fatty acids (FFAs) and cholesterol cause hepatic lipotoxicity and stimulate macrophage activation, and production of proinflammatory cytokines [31]. Palmitate in serum FFAs showed elevation in patients with NASH [32]. Increased levels of palmitic acid and its metabolites, such as phospholipids, diacylglycerol and ceramides, activate LPS-mediated Toll-like receptor (TLR) 4, PKCs, and ER stress and increase ROS production by inducing mitochondrial dysfunction. ER stress and ROS activate the NOD-like receptor pyrin domain containing 3 (NLRP3) inflammasome and NF-κB and increase production of proinflammatory cytokines and chemokines [33]. Moreover, C-C motif chemokine 2 (CCL2, also known as MCP-1), IL-1β, IL-18, and TNF-α recruit bone marrow-derived macrophages to activated hepatic stellate cells and cause damage to the liver. This section is focused on macrophage/Kupffer cells and chemokines, which play an important role in the progression from NAFLD to NASH.

### 2.1. Macrophages and Kupffer Cellsn

Liver macrophages comprise several populations and play a key role in liver immune homeostasis and the pathogenesis of liver disease [34]. Kupffer cells originate from the fetal yolk sack and form a self-renewing pool of organ-resident macrophages, independent of myeloid monocytic cells [35]. Macrophages recruited during inflammation are differentiated from circulating monocytes derived from bone marrow cells [36]. Although the populations of macrophages and their transcriptional controls are adequately characterized in mice, the distinction between macrophage subsets in humans is unclear [37,38,39,40].

Kupffer cells and recruited macrophages regulate liver immune homeostasis and the development of liver diseases. Kupffer cells recruit additional immune cells, including neutrophils and lymphocyte antigen 6C-high (Ly6C^hi^) inflammatory blood monocytes. The latter differentiate to CD11b^+^F4/80^+^ inflammatory macrophages (M1-type), which have phagocytic activity and secrete proinflammatory cytokines, such as TNF-α, IL-6, and IL-1β, and ROS [41,42]. Kupffer cell activation contributes to initial hepatic lipid deposition and liver injury [10]. M1/Kupffer cell-type macrophages secrete inflammatory mediators, such as TNF-α, interleukin (IL)-1β, and IL-6, leading to systemic insulin resistance and NASH [43]. M1 macrophages are stimulated by Toll-like receptor (TLR) ligands, such as lipopolysaccharide (LPS) and interferon-gamma (IFN-γ). By contrast, alternative activation of LY6C^low^F4/80^+^ macrophages (M2-type) with an immunosuppressive, pro-fibrogenic phenotype is observed in the reparative phase of NAFLD and NASH. M2-type macrophages secrete high levels of IL-13 and transforming growth factor-β1 (TGFβ1), resulting in progressive fibrosis [44,45,46]. M2 macrophages ameliorate alcoholic fatty liver disease and NAFLD by inducing apoptosis of M1 macrophages [23]. Therefore, the pathology of NAFLD is associated with dynamic changes in macrophage polarization—M1 macrophages initiate and sustain inflammation and M2 macrophages attenuate chronic inflammation [23,24].

### 2.2. Chemokines and Monocytes

Chemokines recruit and activate monocytes and are important in the progression of chronic inflammation in obesity, which underlies adipose tissue inflammation, insulin resistance, and NAFLD [47]. Chemokines, such as C-C motif ligand (CCL) 2, are produced by KCs and recruited macrophages. CCL2 binds to C-chemokine receptor (CCR), and the resulting CCL2-CCR2 complex induces the recruitment of macrophages into adipose tissue and the liver, leading to hepatic steatosis and insulin resistance in obese patients [48,49]. However, CCL2 deficiency does not affect macrophage infiltration or insulin sensitivity, suggesting that CCL2-CCR2 signaling is not critical for obesity-induced macrophage recruitment or systemic insulin resistance [50,51]. Instead, other chemokines involved in obesity may contribute to macrophage recruitment and insulin resistance. CCR5 deficiency attenuates insulin resistance and hepatic fatty acid accumulation by modulating macrophage recruitment and M1/M2 polarization [52].

There are two major subsets of murine monocytes: differentiation into M1 or M2 macrophages, which is important in the pathogenesis of NAFLD and NASH. These subsets of monocytes are distinguished by expression of CCR2 and CX3C chemokine receptor 1 (CX3CR1) and include CCR2 + Ly6Chi and CX3CR1 + Ly6C- monocytes. Under inflammatory conditions, CCR2 + Ly6Chi monocytes transmigrate and differentiate into M1 macrophages. In steady state, CX3CR1 + Ly6C- monocytes differentiate into anti-inflammatory M2 macrophages and mediate tissue repair [53]. In high-fat (HF) diet-induced obese mice, which exhibit NAFLD features, adipose tissue shows significantly increased macrophage infiltration, inflammation, and tissue remodeling. Furthermore, CCR2 + Ly6Chigh monocytes are recruited to adipose tissue and differentiate into M1 macrophages. CX3CR1 + Ly6C- monocytes accumulate in adipose tissue and are classified as M2 macrophages [54]. DIO mice show increased M1 macrophages and decreased M2 macrophages compared with lean mice, leading to a shift to an M1 dominant macrophage phenotype and inducing inflammation and insulin resistance [52]. Indeed, CCR2 deficiency reduced liver steatosis in DIO mice by suppressing the recruitment of CCR2 + Ly6Chigh monocytes [48,49]. Moreover, loss of CX3CR1 in DIO mice exacerbated insulin resistance, inflammation, and steatohepatitis by inducing a shift to M1 macrophages in adipose tissue [55]. On the other hand, another study using a different strain of Cx3cr1 deficient mice found no changes in obesity-induced inflammation, insulin resistance, and adipose macrophage accumulation, as compared with control mice [56]. The relationship between CX3CR1 expression in immune cells and pathogenesis differs among organs [57,58]. In liver inflammation and fibrosis induced by carbon tetrachloride and bile duct ligation, CX3CR1-deficient mice showed increased accumulation of inflammatory Ly6C+ monocytes [59]. Furthermore, CX3CR1 contributes to hepatic macrophage polarization and ameliorates steatohepatitis by controlling intestinal barrier function and dysbiosis [60]. CX3CR1 and CCR2 may independently regulate monocyte phenotype and macrophage polarization, and contribute to adipose tissue inflammation and insulin resistance, and the progression of NAFLD.

## 3. The Gut–Liver Axis in NAFLD and NASH

The increased prevalence of NAFLD may be linked to increased energy intake caused by dietary changes, such as increased intake of carbohydrate (flour and cereal products), fat, and fructose. Moreover, increased use of corn syrup or fructose as sweeteners and sucralose as a non-caloric artificial sweetener may affect the development of NAFLD [61,62,63]. These changes in diet may alter the gut microbiota, intestinal immune system, and intestinal barrier function, promoting metabolic endotoxemia and low-grade hepatic inflammation, thereby contributing to the development of NAFLD and NASH. Hepatic inflammation due to altered food habits may be attributed to changes in the intestinal environment. Understanding the relationship between diet and the gut–liver axis is important for treating and preventing NAFLD and NASH [64].

### 3.1. Intestinal Permeability and the Microbiota

**Figure 2:**
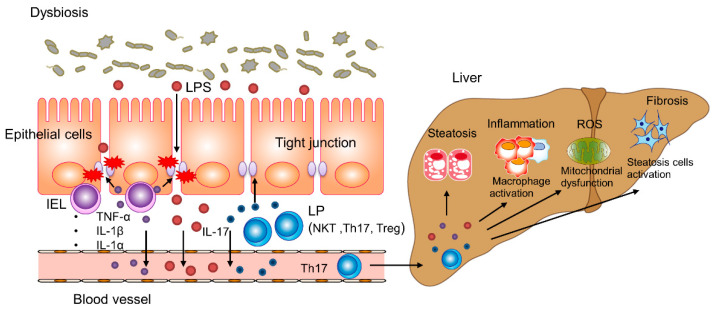
Interaction between dysbiosis-induced alteration of the intestinal mucosal barrier and progression of NASH. Tight junction dysfunction is induced by LPS, and proinflammatory cytokines are produced by intraepithelial lymphocytes (IELs) and the lamina propria (LP), enhancing intestinal epithelial permeability and inducing metabolic endotoxemia followed by hepatic inflammation, steatosis, and fibrosis. Intestinal IL-17 protects tight junctions in the gut, and the serum IL-17 level is increased in NASH patients. The secretion of IL-17 by Th17 cells stimulates monocytes, Kupffer cells, biliary epithelial cells, and stellate cells to secrete proinflammatory cytokines and chemokines, inducing inflammation in the liver.

The liver and gut are impacted by nutrients and the microbiome via the biliary tract, portal vein, and systemic mediators. Liver damage caused by disruption of the gut microbiome, its derived metabolites, and the gut immune system is implicated in the pathogenesis of obesity-induced insulin resistance and NAFLD. The liver is exposed to portal system products, such as pathogen-associated molecular patterns (PAMPs) and damage-associated molecular patterns (DAMPs) and is strongly influenced by diet-induced dysbiosis. PAMPs and DAMPs induce an inflammatory response in hepatocytes, Kupffer cells, and hepatic stellate cells (HSCs) by a Toll-like receptor (TLR) cascade, enhancing release of cytokines and chemokines (such as TNFα, IL-1, IL-6, IL-8, and IFN-γ), resulting in liver damage. Mice fed a HF or choline-deficient diet and patients with NAFLD have increased intestinal permeability [30,65,66], triggering a proinflammatory cascade that worsens hepatic inflammation by facilitating portal influx of microbiome-derived metabolites to the liver [66,67] (Figure 2). Intestinal permeability is regulated by epithelial tight junctions, which consist of several integral membrane proteins, such as zonula occludens (ZO), occludin, junctional adhesion molecule-A (JAM-A), and claudins [68]. Mice fed a HF diet exhibit decreased tight junction proteins and low-grade gut inflammation as a result of microbiome abnormalities. That is, the intestinal barrier and gut vascular barrier are impaired by HF diet-induced microbiome changes, promoting the portal influx of bacterial products, thereby worsening non-hepatic inflammation and metabolic abnormalities (Figure 1). Moreover, the altered microbiota disrupts the intestinal epithelial and vascular barriers by acquiring the ability to cross the intestinal epithelial barrier in mice fed a HF diet [30]. Whether the ability to cross the damaged intestinal epithelium is an active mechanism or a result of increased intestinal permeability caused by decreased expression of tight junction proteins is unknown.

Several clinical studies have suggested a link between the gut microbiota (such as small intestinal bacterial overgrowth and microbial dysbiosis) and the pathogenesis of NAFLD, but causality has not been established [69]. Shotgun metagenomic sequencing indicated an association between a microbiome signature characterized by increased abundance of *Escherichia coli* and *Bacteroides vulgatus* and advanced fibrosis in patients with NAFLD [70]. *Escherichia* abundance was higher in obese children with NASH compared to those with only obesity [71]. Moreover, *Bacteroides* and *Ruminococcus* were significantly increased, and *Prevotella* decreased, in patients with NASH (stage ≥ 2 fibrosis) compared to those without NASH, shown by 16S amplicon sequencing [72]. This finding is consistent with evidence that *Bacteroides* and *Prevotella* are competitors in the gut microbiota, depending on dietary composition [73].

### 3.2. Intestinal Immune System

Intestinal immune cells contribute to the establishment of the intestinal mucosal barrier. These cells are classified as intraepithelial and lamina propria cells. Intraepithelial cells include intestinal intraepithelial lymphocytes (IELs), encompassing several T cell receptor (TCR)-positive and -negative subsets. TCR^+^ and TCR^−^ IELs exhibit different subtypes depending on the developmental conditions: TCR^+^ IELs are induced after antigens are encountered, natural TCRαβ^+^ IELs undergo thymic agonist selection, TCRγδ^+^ IELs differentiate either intrathymically or extrathymically, and the development of TCR^−^ IELs is similar to that of peripheral innate lymphocytes [74,75]. IELs produce proinflammatory type I cytokines (IL-1β, IL-1α, IL-12, TNF-α, and GFAP), are cytolytic, and release antimicrobial peptides upon activation by intestinal epithelial cell-released cytokines or engaging activating natural killer (NK) cell receptors [75]. IELs and intraepithelial mononuclear phagocytes fight infection and induce tolerance to food and microbial antigens. In the lamina propria (LP), immune cells act as a second line of defense and promote regeneration of damaged tissue. The immune cell population of the LP includes T lymphocytes (mostly CD4^+^), NK T lymphocytes, dendritic cells, macrophages, ILCs, IgA^+^ plasma cells, IgG^+^ and IgM^+^ plasma cells, and B lymphocytes [76,77]. CD4^+^ T cells in the LP comprised primarily T helper (Th) 17 cells and regulatory T (Treg) cells. Th17 cells release IL-17A, IL-17F, and IL-22, preventing bacterial dissemination by inducing the expression and secretion of antimicrobial peptides [78]. IL-17A maintained intestinal permeability by regulating the tight junction protein occludin in a dextran sulfate sodium injury model [79]. The reduced proportion of Th17 cells in the small intestine of obese mice induced weight gain and worsened glucose intolerance and insulin resistance [80]. By contrast, IL-17 promoted intestinal barrier dysfunction by disrupting tight junction structure and promoting bacterial dissemination in sepsis models [28]. Notably, the intestinal IL-17 level was elevated in mice with steatohepatitis, and the number of Th17 cells was increased among peripheral blood mononuclear cells in patients with NASH [29,81]. Intestinal Th17 cells migrate to the liver and secrete IL-17 to stimulate monocytes, Kupffer cells, biliary epithelial cells, and stellate cells, and to secrete proinflammatory cytokines and chemokines, triggering inflammation in the liver [82]. The effect of IL-17 in the intestinal tract may differ depending on the disease and is an important therapeutic target for NAFLD and NASH.

## 4. Treatment of NAFLD and NASH with Probiotics, Prebiotics, and Biogenics

Many pharmacotherapeutic strategies have been used for NASH, which can progress to cirrhosis. Insulin sensitizers, such as metformin or pioglitazone, have been studied for the treatment of NASH. In addition, vitamin E, a food ingredient with antioxidant properties, has also been studied for its therapeutic effect on NASH [83,84]. In the TONIC trial (metformin and vitamin E) and the PIVENS trial (pioglitazone and vitamin E), metformin, pioglitazone, and vitamin E improved steatosis and inflammation but not fibrosis. However, pioglitazone led to significant weight gain [85,86]. Clinical and animal studies have evaluated the therapeutic effects on NAFLD and NASH of probiotics, prebiotics and biogenics. These have been shown to ameliorate NAFLD and NASH, but there are issues in developing methods for evaluating clinical studies and appropriate biomarkers for prognosis and diagnosis.

### 4.1. Lactic Fermentation and Control of Gut Microbiome

Studies have investigated the ability of functional foods, such as lactic acid bacteria, to improve the gut microbiome in NASH and NAFLD. Functional foods are classified as probiotics, prebiotics, and biogenics based on their mechanisms of action [87]. Probiotics were defined as “live microorganisms, which when administered in adequate amounts, confer a health benefit on the host” by the 2001 World Health Organization/Food and Agriculture Organization (WHO/FAO) expert consultation. Probiotics positively alter the intestinal microbiota and immune system. Probiotics must promote survival and exert an immunomodulatory effect in the gastrointestinal tract, inhibit pathogenic bacteria, be safe, and not have antibiotic resistance genes [88]. Microorganisms used as probiotics include *Lactobacillus*, *Streptococcus*, *Lactococcus*, *Enterococcus*, *Bifidobacterium*, *Bacillus*, and *Clostridium*. These probiotics promote an anti-inflammatory environment and intestinal epithelial growth and survival and counteract pathogenic bacteria by modulating immunity. Prebiotics were defined by Gibson et al. in 1995 as, “A non-digestible food ingredient that beneficially affects the host by selectively stimulating the growth and/or activity of one or a limited number of bacteria in the colon, and thus improves host health” [89].

Prebiotics include oligosaccharides and dietary fibers. Oligosaccharides encompass fructooligosaccharides and galactoses, such as lactulose, which have highly selective availability to bifidobacteria and promote their growth. The term “synbiotic” refers to a combination of probiotics and prebiotics, as defined by Gibson et al.

Biogenics was proposed by Mitsuoka et al., and applies to food ingredients that contain biologically active peptides and immunopotentiators (biological response modifiers) produced directly or indirectly by modulation of the intestinal microflora [90]. Biogenics encompass various biologically active peptides, plant polyphenols, docosahexaenoic acid, eicosapentaenoic acid, vitamins, and other food components related to biological regulation, biological defense, disease prevention, functional recovery, and aging control. Heat-inactivated probiotic bacteria are also considered biogenics. Probiotics or biogenics are taken up by M cells in Peyer’s patches and transferred to subepithelial dendritic cells, reducing the expression of proinflammatory cytokines in mice [91].

### 4.2. Therapeutic Effects of Probiotics, Prebiotics, and Biogenics on NAFLD and NASH

Several animal model studies and clinical trials have reported evidence of benefits. For example, VSL#3 and modified VSL#3 are a mixture of several probiotic bacteria of the genera *Lactobacillus, Bifidobacterium,* and *Streptococcus*, or *Lactobacillus* alone. VSL#3 protected against insulin resistance and NAFLD by inhibiting inflammatory signaling, such as c-Jun N-terminal kinase (JNK) and nuclear factor-κB (NF-κB) and restoring the reduced number of hepatic natural killer T cells caused by a HF diet [92,93]. By contrast, VSL#3 did not affect methionine-choline-supplemented (MCS) diet-induced hepatic steatosis or inflammation but ameliorated hepatic fibrosis by negatively regulating TGF-β signaling in mice [94]. Other animal model studies showed that probiotics improved the gut microbiota composition and maintained tight junctions, restoring the intestinal mucosal barrier and suppressing serum LPS levels. Liver inflammatory markers (e.g., ALT, AST, hepatic TG, and proinflammatory cytokines) were reduced by a decrease in serum LPS and liver TLR4 mRNA levels [95,96]. *Lactobacillus plantarum* NA136 isolated from fermented food suppressed the body weight gain and decreased the mass of fat tissues of HF diet and fructose-fed mice (NAFLD model).; lipids, AST, and ALT levels were also reduced *L. plantarum* NA136 decreased de novo lipogenesis and increased fatty acid oxidation by activating the AMPK pathway to phosphorylate ACC and suppress SREBP-1/FAS signaling in a NASH model. Furthermore, *L. plantarum* NA136 reduced oxidative stress in the liver by activating the AMPK/NF-E2-related factor 2 (Nrf2) pathway in a NAFLD model. These effects resulted in *L. plantarum* NA136 attenuating NAFLD [97]. Moreover, *Lactobacillus paracasei* decreased the expression of TLR-4, CCL2 and TNF-α and attenuated hepatic steatosis. *L. paracasei* decreased the proportion of M1 Kupffer cells and increased that of M2, leading to M2-dominant shift in the liver of a NASH animal model [98]. The effect of an aqueous probiotic suspension (Symprove^TM^, containing *Lactobacillus acidophilus* NCIMB 30175, *Lactobacillus plantarum* NCIMB 30173, *Lactobacillus rhamnosus* NCIMB 30174, and *Enterococcus faecium* NCIMB 30176) on the composition of human intestinal microbiota was studied using the M-SHIME^®^ system on an in vitro human intestinal model. Three probiotics showed colonization and growth in the luminal and mucosal compartments of the proximal and distal colon, and growth in the luminal proximal colon. This increased the proximal and distal colonic lactate concentrations. Lactate stimulated growth of lactate-consuming bacteria, resulting in increased short-chain fatty acid (SCFA) production, especially butyrate. Additionally, the probiotics exerted immunomodulatory effects, such as increased production of anti-inflammatory cytokines (IL-10 and IL-6) and decreased production of proinflammatory chemokines (IL-8, CXCL 10 and MCP-1) [99]. A meta-analysis of the effects of probiotics on patients with NAFLD/NASH showed improved serum levels of liver aminotransferases, total cholesterol, and TNF-α, and amelioration of insulin resistance. However, the data are difficult to reconcile, given use of different probiotic strains and dosages, treatment durations, and outcome indices [100].

Prebiotics may have a beneficial effect on NAFLD and NASH. In animal models, prebiotics altered the gut microbiota composition and increased the plasma glucagon-like peptide-2 (GLP-2) level, improving gut barrier function. Moreover, prebiotics reduced liver inflammation and improved metabolic disorders in obesity and diabetes [101]. Furthermore, prebiotics including inulin and oligofructose controlled the growth of *Faecalibacterium prausnitzii* and *Bifidobacterium* and reduced the plasma endotoxin level by increasing GLP-1 secretion as well as the GLP-2 trophic effect on gut barrier integrity [102]. In humans, oligofructose supplementation improved glucose tolerance and promoted weight loss by regulating the expression of hormones involved in energy intake, such as ghrelin and peptide YY, in obesity [103]. Moreover, a meta-analysis of probiotic, prebiotic, and synbiotic therapies for NAFLD showed significantly reduced BMI, ALT, and AST. Synbiotics, but not prebiotics or probiotics, did not decrease serum lipids [104].

Heat-killed lactic acid bacteria in biogenics, which are easier to handle compared with live lactic acid bacteria, have been used in studies of NAFLD and NASH. Heat-killed *Lactobacillus reuteri* GMNL-263 (Lr263) reduced fibrosis in the liver and heart by TGF-β suppression in HF diet-fed mice [105]. Similarly, live Lr263 improved inflammation, insulin resistance and hepatic steatosis in high fructose-fed rats [106]. Moreover, heat-killed *Lactobacillus plantarum* L-137 (HK L-137), which is isolated from fermented fish and rice dishes, attenuated adipose tissue and hepatic inflammation in DahlS. *Z-Lepr^fa^/Lepr^fa^* rats as a model of metabolic syndrome [107]. Live and heat-killed *Lactobacillus pentosus* strain S-PT84, isolated from Kyoto pickles (*shibazuke*), reportedly enhances splenic natural killer activity and interferon-γ production in mice [108,109]. Heat-killed S-PT84 partially restored expression of ZO-1, occludin, and xlaudin-3 but did not restore the alteration the microbiota profile in a NASH model. Heat-killed S-PT84 suppressed metabolic endotoxemia by maintaining the gut barrier and intestinal permeability and suppressing IL-17-producing T (Th17) cell accumulation in the intestinal LP. By contrast, heat-killed S-PT84 had no effect on the abundance of NKT cells in the liver. However, heat-killed S-PT84 attenuated hepatic inflammation and fibrosis by decreasing the M1/M2 macrophage ratio in the liver. These results indicated that heat-killed S-PT84 attenuated lipotoxicity-induced hepatic insulin resistance and steatohepatitis in a NASH animal model [110]. By contrast, live *Lactobacillus pentosaceus* LP28 (LP28), isolated from longan fruit (*Euphoria longana*), showed reduced body weight gain, liver triglyceride and cholesterol in HF diet-fed mice. However, heat-killed LP28 did not prevent metabolic syndrome [111]. Heat-killed lactic acid bacteria as biogenics can also improve NAFLD and NASH, as do some live lactic acid bacteria. However, heat-killed lactic acid bacteria will continue to be investigated in clinical and animal studies because of their immunomodulatory effects, long shelf-life, and ease of storage and transportation.

Probiotic, prebiotic, and biogenic treatment of NAFLD and NASH is new and under development, and these agents regulate the gut microbiota and immunity. By contrast, probiotics do not regulate the intestinal environment or improve the symptoms of acute pancreatitis or Crohn’s disease. For instance, administration of *Lactobacillus plantarum* 299 v for at least 1 week preoperatively during the postoperative period in elective surgical patients did not influence bacterial translocation, gastric colonization, or the incidence of postoperative septic morbidity [112,113]. A meta-analysis of six randomized controlled trials involving 536 adults with severe acute pancreatitis showed that probiotics compared with the control did not significantly affect the pancreatic infection rate, total number of infections, operation rate, hospital length of stay, or mortality [114,115]. However, the probiotics did not exacerbate these diseases, and so safety concerns are unlikely. Probiotics, prebiotics and biogenics have been shown to be effective but not curative for NAFLD and NASH. Furthermore, the active components and molecular mechanisms of the therapeutic effects of probiotics, prebiotics and biogenics are unclear, and further studies are needed.

## 5. Conclusions

**Figure 3:**
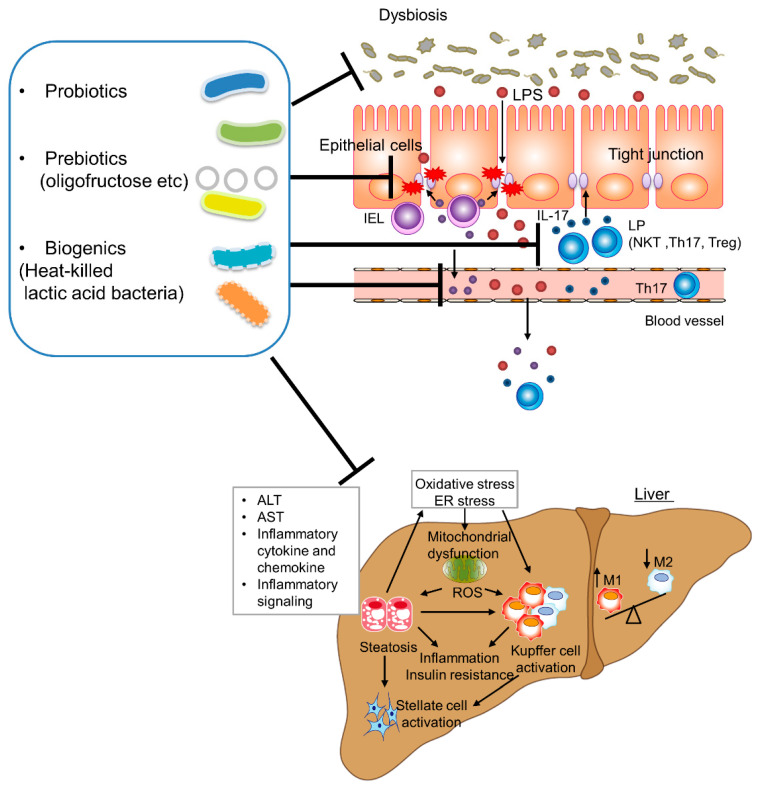
Schematic representation of the beneficial effects of probiotics, prebiotics (e.g., oligofructose), and biogenics (heat-killed lactic acid bacteria) on NAFLD and NASH progression by regulating the intestinal environment and reducing hepatic inflammation, lipid accumulation, and fibrosis. In NAFLD and NASH, probiotics, prebiotics, and biogenics suppress tight junction dysfunction and enhance intestinal epithelial permeability. Increased intestinal permeability or ‘leaky gut’ results in metabolic endotoxemia followed by low-grade inflammation in the liver. Probiotics, prebiotics, and biogenics ameliorate NAFLD and NASH by preventing leaky gut and metabolic endotoxemia due to tight junction dysfunction, and by depressing oxidative stress, inflammation, and fibrosis in the liver.

NAFLD is a common chronic liver disease worldwide, and its prevalence is increasing. Moreover, its association with obesity, type 2 diabetes mellitus, insulin resistance, metabolic syndrome, and progression to cirrhosis and hepatic carcinoma increase its clinical importance. The pathogenesis of NASH and NAFLD is complex, involving not only the hepatic immune system (monocyte or macrophage polarization) mechanisms but also adipokines produced by adipose tissue, and microbiome. Moreover, an altered gut microbiota composition and intestinal immunity are related to liver disease and are important in progression from NAFLD to NASH. In human and animal studies of NAFLD and NASH, probiotics, prebiotics and biogenics reduced serum levels of liver aminotransferases, inflammatory cytokines, and chemokines, and ameliorated insulin resistance and hepatic steatosis (Figure 3). Probiotics, prebiotics, and biogenics ameliorate NAFLD and NASH by regulating the intestinal environment and immunity; therefore, their efficiency is based on the gut–liver axis. Although probiotics, prebiotics, and biogenics are considered safe, their safety should continue to be evaluated. Additionally, future human studies of treatments for NAFLD and NASH should involve standardized probiotic strains and dosages, treatment durations, and outcome indications, as well as make use of advanced techniques, such as omics technologies. Further studies of the efficacy, safety, and molecular mechanisms of probiotics, prebiotics and biogenics in NAFLD and NASH are needed.
